# Evaluating White Worm (*Enchytraeus* sp.) Culture Conditions and Zeolite Supplementation for Aquaculture Live Feed

**DOI:** 10.3390/life15121813

**Published:** 2025-11-27

**Authors:** Meryem Öz, Mehmet Bahtiyar

**Affiliations:** 1 The Department of Aquaculture, Fisheries Faculty, Sinop University, 57000 Sinop, Türkiye; 2 Viasea Aquarium, Tuzla, 34940 Istanbul, Türkiye; mhmtbhtyr@gmail.com

**Keywords:** live feed, white worms, *Enchytraeus* sp., zeolite, aquaculture

## Abstract

This study aimed to determine the culture conditions and some anatomic features for using white worms (*Enchytraeus* sp.) as live feed in aquaculture. In the first experiment (Culture 1), coconut peat was used as the culture media and it was determined that, under approximately 21 °C, the population of the white worm species increased from 10 adult individuals to a total of 424 individuals (including both adults and juveniles) over a 70-day period. Unlike the first experimental stage (Culture 1), the second stage (Culture 2) was structured into three groups. In the second experiment (Culture 2), coconut peat was used as the culture media in the first (G1) and second experimental groups (G2), and zeolite with coconut peat in the third group (G3). The first (G1) and third experimental (G3) groups were fed a control diet, while the second group (G2) was fed a diet with 10% zeolite added to the control diet. In the 60-day study conducted during the culture-2 phase, the worm population, which initially consisted of 10 individuals, reached its highest values in Group 3. The use of zeolite as a substrate and feed additive for white worm culture was investigated for the first time, and original data were obtained that will contribute to filling the gap in the literature. Furthermore, the aquarium filtration sponge used in addition to the culture soil was determined to be a suitable substrate material for collecting cocoon data. The findings of this study provide new supporting data that will fill gaps in the literature regarding materials and methods that contribute positively to white worm culture conditions.

## 1. Introduction

Aquaculture is not only a valuable source of human nutrition but also plays a crucial role in the conservation of species in natural ecosystems.

One of the most important criteria in the cultivation of aquatic organisms is the implementation of optimal nutritional conditions. Nutrition is among the key factors affecting the growth and reproduction of cultured fish, as it enables them to realize their full genetic potential. In pisciculture, in addition to formulated diets, the use of live feed has been shown to positively influence characteristics such as growth, survival and reproduction in fish. One of the most critical criteria for the success of aquaculture is the reliable supply of live feed. In this regard, the production of live feed that can be cultivated in hygienic conditions and is meets the vital needs of aquatic species is of great importance. Juvenile fish, in particular, possess underdeveloped digestive systems and require live, motile prey that can stimulate feeding behavior while fulfilling specific nutritional requirements. Live feeds such as Brachionus (rotifers), Artemia nauplii, copepods, infusoria, and branchiopods provide essential proteins, fatty acids, and micronutrients, in addition to the movement cues necessary for effective prey recognition. White worms represent one such live feed option for fish [1].

In this context, earthworms are considered a high-quality live feed source due to their high protein content and favorable amino acid composition [2,3,4,5].

Among the most suitable species used as live feed are copepods, nematodes, and oligochaetes. Their mass production has not yet been fully developed. It has been reported that further research is needed to enhance knowledge of the biology and physiology of these species in order to optimize mass production and ensure their economic sustainability [6].

The use of white worms produced under controlled conditions as a high-quality live feed has been steadily increasing in both the edible fish sector and in the aquarium fish sector [4,7,8]. Among the species used in aquarium fish cultivation are *Enchytraeus albidus*, *Enchytraeus buchholzi* and *Enchytraeus crypticus*, with *Enchytraeus albidus* being distinguishable from the others due to its relatively larger body size. Although *Enchytraeus albidus* possesses a hermaphroditic reproductive system, individuals exhibit sexual reproduction. During mating, sperm is transferred from one worm to another. The eggs are deposited into a cocoon produced by the clitellum, and these cocoons are typically laid in soil or aquatic environments where the juveniles hatch and develop. In *Enchytraeus albidus*, the egg diameter ranges from approximately 300 μm to 500 μm. In general, sexual maturity is reached within 5 to 7 weeks after hatching [9,10,11,12,13,14,15]. Although the optimal temperature range for embryonic development, body growth, maturation, and reproduction of *E. albidus* has been determined to be between 15 °C and 22 °C, it has been reported that population growth can still occur even at 2 °C and 25 °C. In general, *E. aldibus* is known to survive within a temperature range of −14 °C to +32 °C [6,16].

Over the past 30 years, various culture methods for enchytraeids, particularly *E. albidus,* have been investigated using different media, such as water, soil, and agar. In addition, research aimed at optimizing culture conditions for their use as live feed has increasingly intensified in recent years. The white worm, *E. albidus*, also possesses the potential to be processed and used in dried or frozen form according to demand, thus making it a valuable live feed source for aquaculture. White worm (*E. albidus*) plays a particularly important role in providing essential nutrients required during the growth phase of fish larvae. Moreover, during the late larval developmental stage, in which the larger live feed is a must, it serves as an excellent alternative feed in commercial larviculture [4,7,17,18].

Over the past three decades, the feasibility and sustainability of reducing aquaculture production costs by supplementing diets with highly nutritious worms have been examined in numerous countries. Among these alternative feed sources, oligochaete worms have shown considerable potential owing to their rich nutritional profiles, characterized by high levels of crude protein, essential amino acids, vitamins, minerals, and polyunsaturated fatty acids. Consequently, earthworms, which contain comparable or even greater concentrations of key nutrients, represent promising substitutes for conventional feed ingredients such as squid, krill, and mussel meals. Furthermore, the mass cultivation of these worms in various substrates can influence their nutritional composition, thereby offering a practical avenue for their incorporation into aquaculture diets. Despite their economic relevance, the inclusion of *E. albidus* in aquaculture feeding protocols has been addressed by only a limited number of studies in the literature [4].

Materials such as garden soil, seaweed, peat, and coconut peat were used in white worm culture, but the use of clinoptilolite type zeolite was not observed [6,19]. This study aimed to investigate white worm morphological characteristics and determine the specific effects of zeolite on white worm cultivation.

## 2. Materials and Methods

### 2.1. Experimental Organism and Culture Set Up

In the study, the white worm species *Enchytraeus* sp. was selected as the model organism. *Enchytraeus albidus* was purchased and obtained as a starter culture from local producers. The experimental setup was arranged in accordance [13,16,19,20,21,22,23], with the conditions specified in relevant literature, using polyethylene plastic culture containers, size of 12 × 7 × 9 cm, that are non-toxic and safe for organism health.

### 2.2. Culture and Measurement Instruments

The physical properties of the culture soil used in establishing the experimental setup were measured using a Kagen LCD 4 in 1 model Soil Survey Instrument, while the general properties of the test water were analyzed with a YSI Professional Plus hand-held field and laboratory device. In the experiment, organically produced coconut peat, commercially known as Genta compressed peat, cocopeat, that contained no chemicals or fertilizers, was used. Before use, the soil was dried at 80 °C for 24 h [18,21,22,24]. In addition, natural mineral zeolite, clinoptilolite type, with a particle size of 100 microns was used in the experiment as a regulator of soil and water parameters.

In the experiment, a Labomed Digizoom stereo microscope and a Novex RZ 65500 stereo microscope were used for the observation of cocoons, eggs, and worms. A KERN EW420-3NM precision balance with an accuracy of 0.001 g was used for weighing experimental materials and feed. Additionally, milimetric paper was employed for length measurements.

### 2.3. Innovative Practices

**Figure 1:**
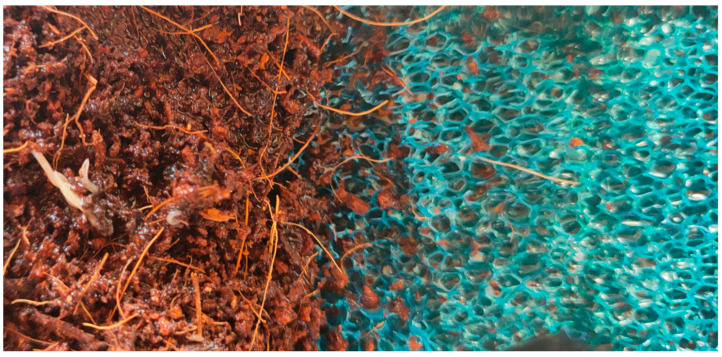
Coconut peat and aquarium internal filter sponge, used in the white worm, *Enchytraeus* sp., culture (Original).

**Figure 2:**
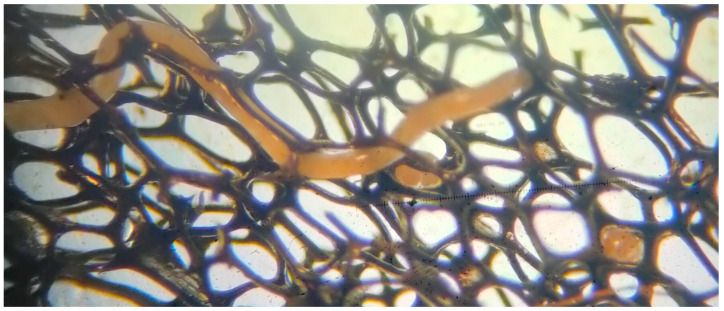
Adult white worm and eggs within cocoons observed on the aquarium internal filter sponge (Original).

To determine the data related to adult worms, cocoons and eggs, an innovative practice not previously reported in the literature was applied, involving the use of aquarium internal filter sponges alongside the culture soil. In this practice, to facilitate the monitoring of cocoons and eggs within them, the amount of soil was reduced by half, and small, 1–2 mm thick pieces of water-resistant aquarium filter sponge, that is non-moldable, resistant to corrosion and non-toxic to organisms, were added. The sponge pieces were examined at frequent intervals to track cocoons and eggs (Figure 1 and Figure 2).

**Figure 3:**
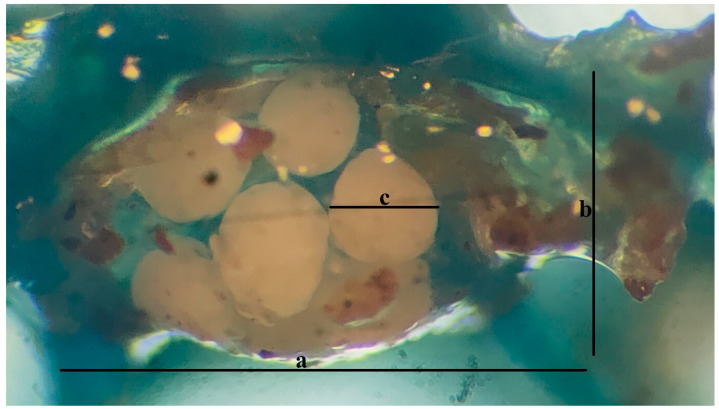
Cocoon and eggs of the white worm, *Enchytraeus* sp. (a: cocoon length, b: cocoon width, c: egg diameter) (Original).

For cocoons, which were generally elliptical in shape, both length and width measurements were recorded. For the transparent, round shaped eggs contained within the cocoons, diameter measures were taken, and the number of eggs per cocoon was determined (Figure 3).

In the experiment, a commercial pond feed formulated for aquarium fish, which contains 31 percent crude protein and five percent fat, was used to feed both adult and juvenile worms during the adaptation, production, and research phases [21,22,25].

### 2.4. Experimental Design

**Figure 4:**
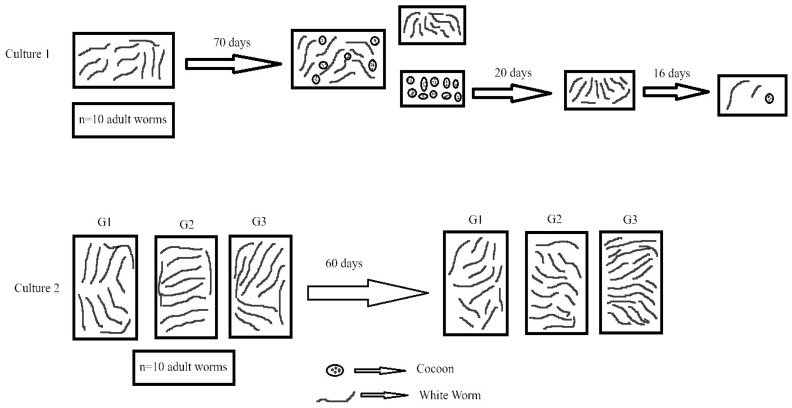
Experimental Setup (It has been drawn based on [26]).

This study was conducted in two culture stages. In both culture stages, ten adult white worms were used at the beginning of the experiment. The experimental setup is shown in Figure 4.

In counting the white worms, a new practice was developed based on techniques used in previous studies and was termed the washing/catching practice. According to this practice, water was gently added to the experimental setup until it slightly covered the soil surface, 1–2 mm above the soil level, and the soil was carefully stirred. The worms, which coiled and clustered on the surface of the water, were then separated from the soil by transferring them along with the water into a separate container. The worms collected into counting container containing a small amount of water were easily counted individually, as they naturally adhered to the tip of a pointed wooden stick due to their attachment reflex. Through this washing application, both adult and juvenile worms were effectively separated from the soil without injury. Their counting was facilitated by their coiling movements in water and was performed with the naked eye, without the need for a microscope. This counting practice differs from the floatation technique reported by [27], as it does not involve the use of dyes or colloidal substances.

### 2.5. Reproductive Monitoring

According to the method reported by [19,27], the number of individuals reproducing in the culture was determined over two consecutive periods. In the initial period, after the initial period of 70 days, all adult and juvenile individuals were separated from the soil medium and counted. During the developmental period, the eggs and larvae present in the cocoons within the culture were allowed to develop. After 20 days, a second count was conducted to determine the total number of the newly hatched individuals.

The development of these new individuals was monitored for signs of sexual maturation and the time to sexual maturity was recorded on day 22, which corresponds to the observation of the first cocoons and juvenile individuals.

In culture stage 2, the effects of clinoptilolite type zeolite as a feed additive and soil conditioner on the white worm culture were investigated. Clinoptilolite zeolite is a natural mineral that contributes to balancing soil and water parameters [28]. The adult white worms (n (number of individuals) = 90) produced in the first culture were used to establish three experimental groups, each consisting of three replicates (Table 1).

In this experimental setup, the first group, G1, served as the control group, in which worms were fed with the control diet without any zeolite added to the feed or soil. In the second group, G2, zeolite was added only to the feed but not to the soil. In the third group, G3, zeolite was incorporated into the soil but not into the feed. In the Culture Stage 2 setup, 10 g of dried coconut peat was used as the culture substrate for the first and second groups. In the third group, the total substrate amount, 10 g, consisted of 5 g of coconut peat and 5 g of powdered zeolite.

The first and third groups were fed with the control diet, while the second group received feed containing 10 percent zeolite mixed into the control diet. After adding the substrate materials to the experimental containers, water was added to achieve a moisture level of 30 to 40 percent, and ventilation holes were made in the lids of the culture containers.

Subsequently, ten healthy looking adult white worms were placed in each replicate [5,16,25,29]. The lids of the containers were closed to maintain internal humidity and to prevent external contaminants or pests from entering the culture environment. The cultures were kept in a dark environment at suitable temperatures (~23.4 **°**C). To maintain moisture levels, the cultures were checked every 2 to 3 days, and dechlorinated water was added when necessary [5,13,16,20,21,22,25,29,30]. Soil parameter measurements, feeding, aeration, and moisture regulation, as well as observations of adults, juveniles, cocoons, and eggs were performed through routine monitoring. At the end of the experimental period, all individuals, both adults and juveniles, in the culture environment were counted alive to determine the increase in the white worm population. It has been reported that fixed worms are shorter than live ones due to contraction after fixation [31]. Length measurements of the worms were taken in moistened glass Petri dishes placed on millimetric paper by observing the natural movement of live white worms.

### 2.6. Statistical Analysis

All data are presented as mean ± standard error (SE). Minitab Statistical Software for Windows (release 17) was used for statistical analyses. Means were compared with one-way analysis of variance (One-way ANOVA), and differing groups were determined with Tukey’s HDS test. The confidence interval was set to 95%.

## 3. Results

In this study, the culture conditions of the *Enchytraeus* sp. species were investigated due to its potential as a high-quality nutritional source for aquaculture.

At the beginning of the experiment, the pH value of the moistened peat was adjusted to 7.02. Throughout the experimental period, the temperature, humidity, and pH parameters of the culture medium were measured weekly and were determined to be, on average, 20.75 ± 0.53 °C, %42.50 ± 1.64, and 6.81 ± 0.09 for Culture 1, respectively. For Culture 2, the mean values of the parameters recorded over a two-month period are presented in Table 2.

During Culture 1, the increase in the number of individuals in the culture medium was determined and is presented in Table 3. After 70 days, the culture was examined, and the total number of individuals was recorded. All individuals were then removed from the medium and a 20-day period was allowed for the development of individuals emerging from the cocoons. After this period, a second count was conducted, and the total number of individuals was determined to be 751 at the end of 90 days. Subsequent observations of the culture indicated that the first cocoon formation and the appearance of the first juvenile worm occurred on day 22, marking the onset of sexual maturity.

At the end of the experiment, the lengths of the adult individuals obtained ranged between 14 and 27 mm and their diameters were measured between 0.3 and 0.6 mm.

**Figure 5:**
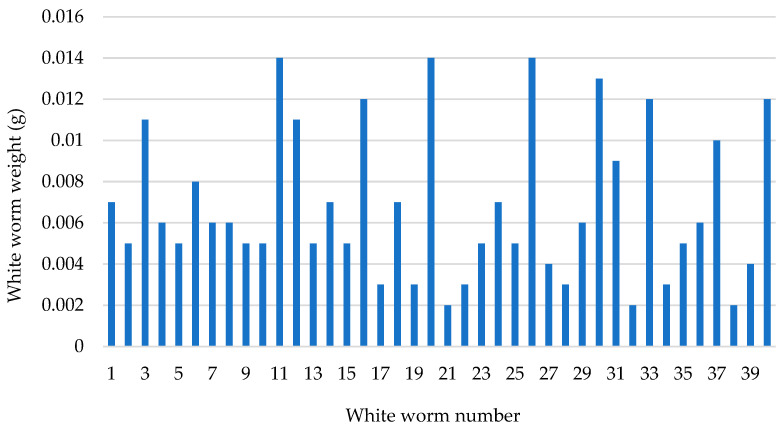
Weight distribution of the white worm population.

At the end of 70 days, a total of 424 juvenile and adult white worms were obtained, of which 40 individuals were randomly selected. The mean live weight of the individuals was determined to be 0.0068 ± 0.00 g (Figure 5).

The hatching period from cocoons was monitored at two different temperatures. At 23 °C (n = 3), the mean duration was 7.67 days. At 19 °C (n = 3), it averaged 12.33 days. The lengths of the juveniles emerging from the cocoons ranged between 0.5 and 1 mm. In this culture, aquarium internal filter sponges were used, and since the worms deposited their cocoons on these sponge pieces, the cocoons could be easily and safely removed from the environment and examined under a microscope.

A total of ten cocoons obtained from Culture 1 were measured for length and width as follows:

1st cocoon: 1200/600 µm, 2nd cocoon: 1100/800 µm, 3rd cocoon: 990/780 µm, 4th cocoon: 1236/1051 µm, 5th cocoon: 800/670 µm, 6th cocoon: 1000/700 µm, 7th cocoon: 1200/1100 µm, 8th cocoon: 1000/650 µm, 9th cocoon: 800/800 µm, and 10th cocoon: 930/700 µm.

Diameter measurements of ten eggs revealed a minimum of 290 µm and a maximum of 530 µm. The number of eggs per cocoon ranged from a minimum of 4 to a maximum of 17.

The data obtained at the end of the second culture stage, which was conducted with three different experimental groups, are presented in Table 4.

At the end of the Culture 2 experiment, the minimum mean increase in the number of individuals was recorded in the control group, with 58.67 ± 4.67 individuals, while the maximum mean increase was observed in the group containing zeolite in the substrate, with 134 ± 3.79 individuals (*p* < 0.05). In both Culture 1 and Culture 2, the practice referred to as the catching technique used for counting white worms was detected to be simple, non-destructive, and effective in its application.

## 4. Discussion

At the end of this study, after 70 days of culture, the adult individuals were removed from the environment, and following an additional 20-day period, the newly developed individuals emerging from the eggs inside the cocoons were counted, totaling 327. The total number of newly produced individuals after 90 days was determined to be 751, representing an increase from the initial 10 individuals to 751. In other words, the population increased approximately 75-fold. The time required for the new individuals to reach maturity was determined to be 22 days at approximately 24 °C. Similarly, in a study conducted by [12], it was reported that enchytraeid worms reached sexual maturity 5–7 weeks after hatching. In the same study, it was stated that in the culture of *E. albidus*, adult worms were separated and counted after three weeks, and following the removal of adults, the experiment continued for another three weeks to determine the number of individuals hatched from the cocoons. Furthermore, the researchers detected that the highest number of juveniles averaged 142 individuals, while the lowest number was 51 individuals. Ref. [32] separated and counted adult worms from the *E. albidus* culture after three weeks. The culture conditions were then maintained for an additional three weeks to allow cocoon development, and at the end of this period, the soil was divided into small portions, and the number of juveniles was determined under a microscope. The researchers reported that the average number of juveniles per culture container was 50, with a maximum of 67 individuals. Ref. [33] stated that the survival and maturation periods of *E. albidus* were influenced by temperature, noting that sexual maturity was reached in 20 days at 25 °C and in 30 days at 15 °C. The life cycle of *E. albidus* is relatively short. It reaches sexual maturity in 33 days at 18 °C and in 74 days at 12 °C [27,34].

In this study, examination of the cocoon and egg findings revealed that the cocoons, which were generally elliptical in shape, had lengths and widths ranging between a minimum of 800/670 µm and a maximum of 1236/1051 µm. The diameter of the circular eggs within the cocoon ranged from a minimum of 290 µm to a maximum of 530 µm, and the number of eggs within the cocoon ranged from a minimum of 4 to a maximum of 17. The hatching period from the cocoon was determined to average 7.67 days at 23 °C and 12.33 days at 19 °C. Similarly, [17] reported that the number of eggs per cocoon as 7 and the cocoon length as 1 mm. Ref. [11] detected egg diameters ranging between 300 and 500 µm, while [25] reported that cocoons of young individuals contained 9 to 10 eggs, whereas this number increased to 20 to 25 in mature individuals, and decreased to 2 to 3 in older individuals, with the maximum number of eggs in a cocoon recorded as 35. Furthermore, in *E. albidus*, hatching from the egg occurs within 12 days at 20 °C and reproduction begins on approximately the 20th day.

An individual *E. albidus* can live up to 9 months and produce as many as 50 cocoons, each containing between 3 and 20 viable eggs. The embryonic development period of the white worm has been reported to be approximately 18 days at 12 °C. Higher temperatures have been shown to cause adults to mature earlier, within 60 days at 9.8 °C and 15.1 °C, and within 40 days at between 17.2 °C to 24.9 °C, and to accelerate the emergence of second-generation juveniles [6,17,35,36]. The embryonic development of eggs inside cocoons has been reported to last approximately 16 days at 18 °C, after which the juveniles hatch [16].

In this study, after a 70-day experimental period (Culture 1), the adult individuals of *Enchytraeus* sp. were detected to have body lengths ranging from 14 to 27 mm and diameters between 0.3 mm and 0.6 mm. Similar values have been reported in previous studies. Ref. [10] recorded lengths of 10 to 35 mm and diameters of 0.7 to 1 mm. Ref. [17] observed adult lengths of 15 to 35 mm and newly hatched individuals measuring 1 to 2 mm. Ref. [18] reported lengths of 15 to 25 mm for adults and 2 to 4 mm for juveniles.

In the studies conducted by [14,18,22,24,27,30,36,37,38,39], various methods were employed, including heat or light treatments, staining, washing, partial counting, and microscopic or image-based counting techniques. In contrast, the washing/catching practice used in this study for counting white worms was newly applied by the authors. Unlike previous approaches, this practice enabled counting and morphometric measurements to be performed easily and non-destructively, without the use of chemicals or costly equipment.

Unlike previous studies [9,40,41], aquarium internal filter sponge pieces were used for the first time in this study to determine cocoon and egg data. It was observed that these sponge pieces were strongly preferred by white worms as a surface for cocoon attachment. Therefore, it was concluded that in future studies, the use of such sponge materials in culture environments could be recommended for ease of cocoon monitoring, as well as for the safe transfer and microscopic examination of cocoons without causing damage.

In the Culture 2 experiment, conducted over a 60-day period at approximately 24 °C, the number of *Enchytraeus* sp. individuals that started with 10 worms reached mean values of 58.67 ± 4.67, 71.67 ± 2.96 and 134 ± 3.79 individuals in Groups 1 (Control), 2 and 3, respectively. Zeolite added to the soil is more effective in white worm reproduction than zeolite added to the feed. Therefore, worm productivity was higher in G3. This may be due to the direct mixing of zeolite with the soil, the duration of contact, and the amount. Similarly, [25] reported that in a 91-day study with *Enchytraeus albidus*, the number of individuals increased from 10 at the start of the experiment to 342 in the group showing the best performance. Ref. [21] investigated *E. albidus* cultures using different feeds and substrates and detected that, starting with 150 worms weighing 0.011 g each, the number of individuals after 50 days ranged from a minimum of 57 to a maximum of 2220.

The use of clay mud, sand silt, and mangrove mud as substrates for the *Nereis virens* (marine worm) was investigated, and mangrove mud was determined to be the ideal substrate with the highest growth and survival rates for the worms. It was also reported that the substrate type could affect the nutritional composition of this species [42]. The specific crystal structure of clinoptilolite provides the necessary microporosity required to increase the ability to retain water, whereas the secondary mesoporosity formed through the connection between clinoptilolite particles and other minerals in the zeolite rocks provides adequate aeration and drainage conditions, making clinoptilolite an idealized inorganic amendment to improve the physical and chemical properties of the soil [43].

When examining the effects of zeolite application on culture conditions, no significant differences were observed among the experimental groups in terms of temperature, approximately 23.4 °C, or humidity, ranging between 40 percent and 42 percent. This may be attributed to the general pH-increasing properties of zeolite. However, statistically significant differences were detected in pH values. The use of zeolite in the culture soil (G3) and in the feed (G2) contributed to slightly more alkaline pH levels, with values recorded as 6.89 in the control group, 7.03 in the group with zeolite in the feed, and 7.22 in the group with zeolite in the soil. Ref. [13] reported that the pH of worm cultures was influenced by both the feeding regimen and the culture process, leading to an increase in acidic conditions. Similarly, [29], as well as [19,44], noted that the use of different substrate materials in worm culture environments affected individual population growth, cocoon production and pH values.

The culture environment of the white worm should be slightly acidic, as required by the species’ biological characteristics. However, studies on culture conditions have reported that, over time, the soil structure tends to deviate from optimal limits, necessitating periodic renewal or the addition of CaCO_3_ (lime, calcium carbonate) to maintain suitable conditions [17,27,29,44,45].

Zeolites are natural materials that have long been used to regulate water and soil properties [28]. Zeolite is mildly alkaline rather than acidic, and when used together with fertilizers, it can help buffer soil pH levels, thereby reducing the need for lime application [46].

Similarly to the present study, no previous search has been conducted regarding the use of zeolite in white worm cultivation. In this context, the positive results obtained are of original value and it has been determined that the use of zeolite in white worm culture environments can be recommended. It has also been reported that white worm cultures require renewal after a certain period of time. Future studies could focus on investigating the growth rate of the culture and determining optimal intervals for renewing the culture medium.

The internal filter sponge, which is specifically designed for aquatic organisms and characterized by its hygienic, resistant-to-corrosion, non-moldable and porous structure, has been proven to be suitable for use alongside soil as both a habitat and a breeding substrate for the worms.

## 5. Conclusions

The use of zeolite in combination with culture soil resulted in a significant increase in the number of individuals in worm cultures compared to the groups that used cocopeat alone. This study demonstrates that zeolite can be recommended as an additive for white worm culture. The application of zeolite as both a substrate and a feed additive in white worm culture media has been investigated for the first time and original data have been obtained.

A new counting technique, adapted from previously reported methods, adapted from a previously reported method, was successfully tested for counting worms. In addition, the use of aquarium filtration sponges alongside culture soil was detected to be a suitable substrate material for collecting cocoon data. In this respect, the study provides novel findings. Overall, the results contribute valuable evidence to fill existing gaps in the literature regarding materials and methods that positively influence white worm culture conditions.

## Figures and Tables

**Table 1:** Use of clinoptilolite type zeolite as soil conditioner and feed additive in culture 2.

	Fish Feed	Soil
G1	None	None
G2	+	None
G3	None	+

**Table 2:** Parameters determined during Culture 2 (mean ± SE).

Culture 2 Parameters *	Group 1 (Control)	Group 2	Group 3
Temperature (°C)	23.44 ± 0.28	23.39 ± 0.26	23.39 ± 0.22
pH	6.89 ± 0.05 ^b^	7.03 ± 0.05 ^b^	7.22 ± 0.05 ^a^
Moisture (%)	42.22 ± 1.73	41.67 ± 1.67	40.00 ± 1.40

* Means followed by different superscript letters within the same line are significantly different (*p* < 0.05).

**Table 3:** Mean initial length and weight of adult white worm at the beginning of the trial, final total weight and number of new individuals hatched from cocoons in Culture 1.

Adult Worms Mean Length (mm) (n = 10)	Adult Worms Mean Weight (g) (n = 10)	Total Weight (g) of Individuals (n = 424) on Day 70	Numbers of New Individuals Hatched from Cocoons on Day 90 (Number) *
24.30 ± 1.56	0.0123 ± 0.00	2.54	327

* Number of individuals hatched from cocoons 20 days after the removal of adults and juvenile from the culture.

**Table 4:** Mean number of individual data determined after 60 days in the experimental groups of Culture 2.

Experimental Groups *	Mean Number of Individuals at the End of the Experiment
Group 1	58.67 ± 4.67 ^b^
Group 2	71.67 ± 2.96 ^b^
Group 3	134 ± 3.79 ^a^

* Means followed by different superscript letters within the same column are significantly different (*p* < 0.05).

## Data Availability

The original contributions presented in the study are included in the article, further inquiries can be directed to the corresponding author.
